# Textural features in pre-treatment [F18]-FDG-PET/CT are correlated with risk of local recurrence and disease-specific survival in early stage NSCLC patients receiving primary stereotactic radiation therapy

**DOI:** 10.1186/s13014-015-0407-7

**Published:** 2015-04-22

**Authors:** Thomas Pyka, Ralph A Bundschuh, Nicolaus Andratschke, Benedikt Mayer, Hanno M Specht, Laszló Papp, Norbert Zsótér, Markus Essler

**Affiliations:** Nuklearmedizinische Klinik und Poliklinik, Klinikum rechts der Isar der TU München, Ismaninger Str, Munich, Germany; Klinik und Poliklinik für Nuklearmedizin, Rheinische Friedrich-Wilhelms-Universität Bonn, Sigmund-Freud-Straße, Bonn, Germany; Klinik für Strahlentherapie und Radiologische Onkologie, Klinikum rechts der Isar der TU München, Ismaninger Str, Munich, Germany; Klinik für Radio-Onkologie, UniversitätsSpital Zürich, Rämistrasse, Zurich, Switzerland; Mediso Medical Imaging Systems, Alsotorokvesz, Budapest, Hungary

**Keywords:** Stereotactic body radiation therapy, NSCLC, FDG-PET, Textural analysis

## Abstract

**Background:**

Textural features in FDG-PET have been shown to provide prognostic information in a variety of tumor entities. Here we evaluate their predictive value for recurrence and prognosis in NSCLC patients receiving primary stereotactic radiation therapy (SBRT).

**Methods:**

45 patients with early stage NSCLC (T1 or T2 tumor, no lymph node or distant metastases) were included in this retrospective study and followed over a median of 21.4 months (range 3.1–71.1). All patients were considered non-operable due to concomitant disease and referred to SBRT as the primary treatment modality. Pre-treatment FDG-PET/CT scans were obtained from all patients. SUV and volume-based analysis as well as extraction of textural features based on neighborhood gray-tone difference matrices (NGTDM) and gray-level co-occurence matrices (GLCM) were performed using InterView Fusion™ (Mediso Inc., Budapest). The ability to predict local recurrence (LR), lymph node (LN) and distant metastases (DM) was measured using the receiver operating characteristic (ROC). Univariate and multivariate analysis of overall and disease-specific survival were executed.

**Results:**

7 out of 45 patients (16%) experienced LR, 11 (24%) LN and 11 (24%) DM. ROC revealed a significant correlation of several textural parameters with LR with an AUC value for entropy of 0.872. While there was also a significant correlation of LR with tumor size in the overall cohort, only texture was predictive when examining T1 (tumor diameter < = 3 cm) and T2 (>3 cm) subgroups. No correlation of the examined PET parameters with LN or DM was shown.

In univariate survival analysis, both heterogeneity and tumor size were predictive for disease-specific survival, but only texture determined by entropy was determined as an independent factor in multivariate analysis (hazard ratio 7.48, p = .016). Overall survival was not significantly correlated to any examined parameter, most likely due to the high comorbidity in our cohort.

**Conclusions:**

Our study adds to the growing evidence that tumor heterogeneity as described by FDG-PET texture is associated with response to radiation therapy in NSCLC. The results may be helpful into identifying patients who might profit from an intensified treatment regime, but need to be verified in a prospective patient cohort before being incorporated into routine clinical practice.

## Background

Hypofractionated stereotactic body radiation therapy (SBRT) has been shown to be a safe and potentially curative treatment option in patients with early stage non-small cell lung cancer (NSCLC) [1-3] and is especially attractive for patients with a compromised health status not eligible for surgery. Excellent local control rates of over 90% – rivaling surgery – have been reported, while overall survival is considerably lower compared to patients receiving primary tumor resection, due to significant comorbidities. Predictive recognition of therapy failure would be favorable, as this might allow options for treatment intensification such as simultaneous or adjuvant chemotherapy, or immunotherapy. However, identification of tumor recurrence through follow-up computed tomography (CT) scans is difficult, as imaging changes due to tumor remnants or radiation reaction may be visible for prolonged periods after treatment. Similarly, early post treatment positron emission tomography (PET) with ^18^ F-fluorodeoxyglucose (FDG) suffers from limitations, because radiation-induced reactive changes such as radiation pneumonitis can cause unspecifically elevated tracer uptake [4,5]. Some authors suggest that follow-ups should be delayed until 12 months after initial therapy in order to reach a sufficient specificity [6,7]. Therefore, the development of surrogate markers for tumors prone to early recurrence is of particular interest. In recent studies, maximum uptake of FDG in pre-treatment PET scans showed a moderate ability to predict overall survival or local recurrence [8-10], but the relation is weak and has been questioned in other publications [11,12].

In the recent years, the measurement of spatial heterogeneity by methods described as “textural analysis” has gained attendance as a means to extract predictive information from FDG-PET scans of several tumors, including sarcoma [13], head and neck tumors [14] and esophageal carcinoma [15]. In NSCLC, a histogram-based heterogeneity parameter has been proposed, but has not been validated on clinical data [16]. More recently, good results have been reached with higher-dimensional heterogeneity features, which have been associated with response and survival after radiochemotherapy in NSCLC [17,18].

In this study, we wanted to verify these promising results in patients with early stage NSCLC who received SBRT as the sole primary treatment modality. Patients included were considered unsuitable for surgery due to concomitant disease, mainly affecting pulmonary and cardiac function. In accordance to earlier publications, textural features derived from histogram analysis, neighborhood gray-tone difference matrices (NGTDM) and gray-level co-occurrence matrices (GLCM) were investigated [19]. We hypothesized that these parameters may be associated with local recurrence, mediastinal lymph node or distant metastases, as well as with overall or disease-specific survival. The results were compared to ‘classical’ PET parameters like maximum and mean FDG-uptake and metabolic tumor volume (MTV).

## Methods

### Patients

Forty-five consecutive patients with histologically proven lung malignancy treated with primary SBRT were analyzed retrospectively. All patients had early stage tumors (T1 or T2, N0, M0), but were considered not eligible for surgery due to concomitant disease after discussion in an interdisciplinary tumor board. All patients received FDG-PET/CT scans before treatment. Written informed consent was obtained before each PET scan as part of the clinical routine. Retrospective analysis of the data was approved by the local ethical review board of the Klinikum rechts der Isar der Technischen Universität München.

### PET/CT studies

^18^ F-FDG PET/CT scans were obtained before start of treatment using a Biograph 16 PET/CT scanner [20]. To achieve standardized metabolic conditions, patients fasted for a minimum of 6 h prior to scanning. Blood glucose level was <150 mg/dl in all patients before injection. Studies comprising 6–7 bed positions each for 3 min were acquired about 60 min after injection of the tracer. Patients in general received low-dose CTs (24–26 mAs, 120 kV) for attenuation correction, as contrast enhanced CTs for morphological correlation were available for all patients before the examination. Data was reconstructed iteratively using the ordered subset expectation maximization algorithm (OSEM) implemented by the manufacturer including scatter and attenuation correction based on the CT data using 4 iterations and 16 subsets. The images were reconstructed into 164 × 164 matrices with a resulting voxel size of 4.06 by 4.06 mm and a slice thickness of 5.0 mm.

### Image analysis

Image analysis was performed with InterView Fusion (Mediso Medical Imaging Systems, Budapest, Hungary). Tumor volumes (volumes of interest - VOIs) were defined by a 3D standardized uptake value (SUV) 2.0 isocontour around the hottest voxel, with manual corrections where necessary. For comparison, VOIs based on SUV 2.5 isocontours were also defined. The maximum SUV (SUV_max_), mean SUV (SUV_mean_), metabolic tumor volume (MTV), and coefficient of variation (COV) were calculated on floating-point SUV data. The SUV used for calculations was the measured activity concentration normalized to decay corrected injected activity and body weight of the patient. For determination of textural parameters, SUV values were normalized and discretized to a total of 64 bins by the formula

$$ R(x)=64\times \left[I(x)-SU{V}_{min}\right]/\left[SU{V}_{max}-SU{V}_{min}\right] $$

where I(x) is the SUV of voxel x in the original image and R(x) is its resampled value. Subsequently, an analysis via neighborhood gray-tone difference matrices (NGTDM) and gray-level co-occurence matrices (GLCM) in 13 directions (3D) was carried out as described earlier [15]. From these matrices, local entropy, correlation, contrast, coarseness and busyness were derived. Additionally, tumor diameters were measured in the low-dose CT image.

### Radiation treatment and follow-up

Stereotactic radiation therapy was curative in intent and delivered using a hypofractionated scheme. Treatment technique and delivery has been previously reported [21]. Briefly, patients were immobilized in a vacuum couch and a free-breathing planning CT (either as slow CT or as 4D-CT) was acquired. In lung window mode, gross tumor volume (GTV) was delineated and an internal target volume (ITV) generated according to the breathing pattern of the patient. Finally, an isotropic margin of 5 mm axially and 1 cm craniocaudally was added to generate the planning target volume (PTV). The total administered dose was 24–45 Gy delivered in 3–5 fractions. Dose was prescribed to the 60% isodose line which had to cover the PTV entirely. Tumors measuring less than 5 cm were treated with 3 fractions of 10–15 Gy (Dmax 16.7 Gy-25 Gy). Larger (>5 cm) or central tumors received 4–5 fractions of 7–8 Gy (Dmax 11.7 Gy – 13.3 Gy). After treatment, follow-up examinations were scheduled in intervals of 3–4 months, including chest CT scans.

### Statistical analysis

The above-mentioned parameters were tested for their ability to predict local recurrence (LR), mediastinal lymph node metastases (LN) and distant metastases (DM) using receiver operating characteristic (ROC). ROC analysis was also used to determine thresholds for survival analysis. Decision thresholds were considered optimal when the Euclidian distance between the ROC curve and the left upper corner of the graph reached the minimum. In addition, for each method the total area under the curve (AUC) was calculated. For local recurrence, disease-specific (DSS) and overall survival (OS), Kaplan-Meier curves were estimated and distributions of survival times were compared between groups using the log-rank test. Univariate and Multivariate Cox regression was used for estimation of hazard ratios (HRs) with 95% confidence interval (CI). SPSS 22 (IBM Inc., Armonk, NY) was employed for statistical analysis. A two-sided level of significance of 5% was used for all tests.

## Results

Patients comprised 24 adenocarcinomas and 18 squamous cell carcinomas. For 3 patients, histology was inconclusive but small-cell lung cancer could be excluded. Detailed patient data is given in Table 1.

**Table 1:** **Patient characteristics**

**Patient characteristic**	**Value**
Age (years)	74 ± 8.1
Sex (n)	
Male	14 (31%)
Female	31 (69%)
T-Stage (n)	
T1	15 (33%)
T2	30 (67%)
Histology (n)	
Squamous	24 (53%)
Adeno	18 (40%)
Other	3 (7%)
Location (n)	
Central	14 (28%)
Peripheral	31 (61%)
GTV (cc)	40.6 (4.2–153)
Movement amplitude (mm)	4.7 ± 2.9*
Tumor size (mm)	
T1	24.0 (17.0–30.0)
T2	44.4 (31.0–68.0)
KPS	80 (60–100)
Time of diagnosis	2005–2010
Follow-up (months)	21.4 (3.1–71.1)
Number of deaths (n)	28
Attributed to tumor	12
Other causes	16

GTV - gross tumor volume; KPS - Karnofsky Performance Scale; *obtained by 8 of the patients who had 4D-CT.

### Local recurrence

**Figure 1:**
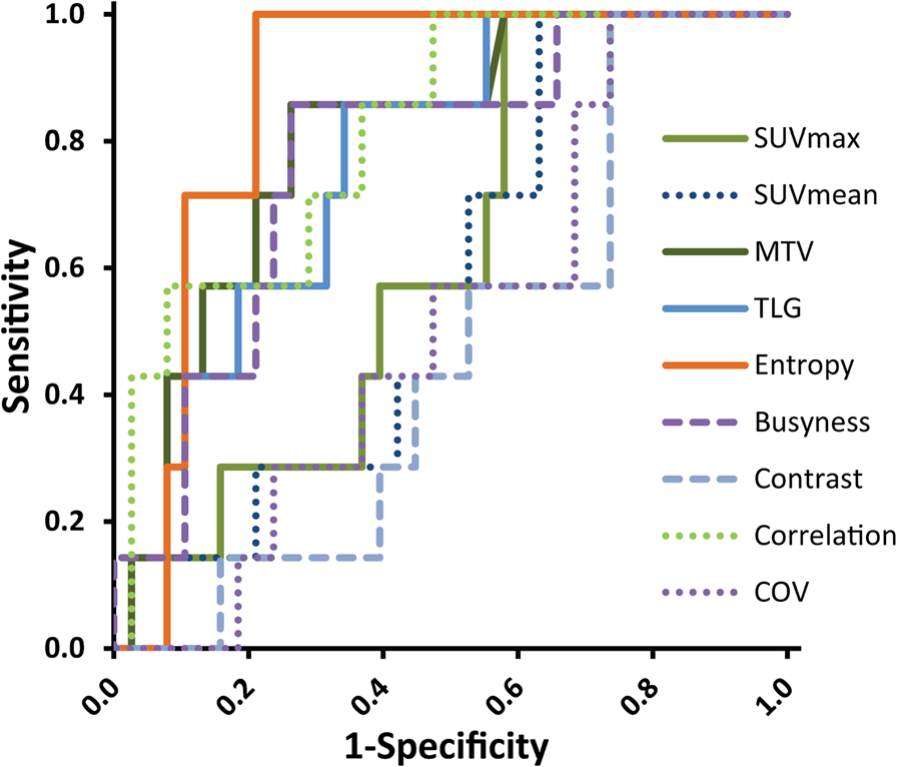
Value of textural and standard PET parameters for prediction of local recurrence. ROC curves for prediction of local recurrence through different PET parameters. Coarseness is the same curve as busyness.

LR was observed in 7 patients. Several textural parameters of heterogeneity, namely entropy, correlation, busyness and coarseness were able to predict LR on a significant level. Areas under the ROC curve were 0.872 (0.770–0.974), 0.816 (0.663–0.969), 0.774 (0.602–0.946) and 0.774 (0.602–0.946), respectively (see Table 2, Figure 1). MTV and tumor size measured in CT were also predictive, with AUC values of 0.806 (0.652–0.960) and 0.739 (0.588 - 0.889) in the whole patient cohort. In contrast, no significant associations between SUV_max_, SUV_mean_ or COV and LR could be shown.

**Table 2:** **ROC statistics for prediction of recurrence and survival by PET parameters**

**Parameter**	**LR**	**LR T2**	**LN**	**DM**	**DSS**	**OS**
Entropy	**.872 (.770 - .974)**	**.801 (.646 - .956)**	.656 (.476 - .837)	.654 (.458 - .849)	**.729 (.545 - .913)**	.664 (.496 - .855)
Correlation	**.816 (.663 - .969)**	**.776 (.589 - .964)**	.586 (.362 - .809)	.572 (.366 - .778)	.680 (.503 - .856)	**.685 (.514 - .855)**
Contrast	.466 (.272 - .661)	.429 (.205 - .652)	.528 (.362 - .809)	.532 (.344 - .720)	.490 (.311 - .688)	.408 (.226 - .589)
Busyness	**.774 (.602 - .946)**	.702 (.487 - .917)	.528 (.335 - .721)	.564 (.362 - .766)	.630 (.432 - .828)	.540 (.359 - .721)
Coarseness	**.774 (.602 - .946)**	.702 (.487 - .917)	.473 (.362 - .809)	.436 (.234 - .638)	.370 (.172 - .568)	.460 (.279 - .641)
SUV_max_	.613 (.428 - .797)	.559 (.351 - .767)	.636 (.456 - .817)	.631 (.423 - .839)	.612 (.426 - .798)	.517 (.335 - .699)
SUV_mean_	.575 (.386 - .764)	.516 (.301 - .730)	.678 (.491 - .864)	.658 (.464 - .851)	.633 (.451 - .814)	.511 (.325 - .695)
COV	.519 (.322 - .715)	.460 (.244 - .676)	.608 (.398 - .818)	.547 (.279 - .814)	.544 (.362 - .726)	.471 (.286 - .655)
MTV	**.806 (.652 - .960)**	.736 (.541 - .931)	.557 (.372 - .743)	.582 (.374 - .789)	.676 (.479 - .872)	.630 (.454 - .806)
CT diam.	**.739 (.588 - .889)**	.568 (.347 - .790)	.547 (.365 - .728)	.541 (.352 - .731)	.630 (.452 - .808)	.620 (.437 - .803)

Shown are AUC values with CI. LR, LN, DM - local, mediastinal lymph node and distant recurrence in all patients; LR T2 - local recurrence in T2 subgroup (diameter > = 3 cm).

Significant values are printed in bold.

As these results suggested a strong dependence of local recurrence on tumor size, we divided the cohort further into T1 (diameter < = 3 cm) and T2 subgroups (>3 cm). All 7 cases of LR were observed in the T2 group, with entropy and correlation still being able to predict LR significantly, while busyness, coarseness, SUV_max_, MTV and CT tumor size were no longer predictive (Table 2). AUCs were 0.801 (0.646 - 0.956) for entropy and 0.776 (0.589 - 0.964) for correlation.

Time to local recurrence was compared between low- and high-risk groups as determined by ROC analysis. Again, entropy and correlation showed significant correlations (p < .001 and p < .004) in the log-rank test. Busyness also revealed a weak, but significant correlation with time-to-LR (p < .042). The remaining PET parameters and tumor size were not predictive.

LN and DM occurred in 11 patients, respectively and were not significantly correlated to any of the examined conventional or textural PET parameters. The highest AUCs were found for SUV_mean_, yielding 0.678 (0.491 - 0.864) for LN and 0.658 (0.464 - 0.851) for DM.

### Disease-specific and overall survival

**Figure 2:**
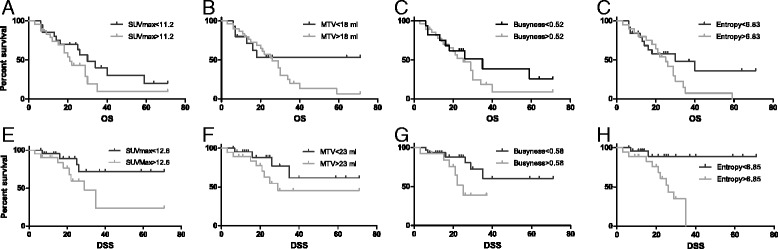
Survival curves for OS and DSS stratified to different PET parameters. Overall and disease-specific survival of subgroups determined by SUV_max_ **(A and** **E)**, MTV **(B and** **F)**, busyness **(C and** **G)** and entropy **(D and** **H)** are shown.

Median follow up was for 21.4 months (range 3.1–71.1). In ROC analysis, entropy was predictive for disease-related death which occurred in 12 individuals, while the feature correlation was significantly associated with death due to any cause (28 events). No other significant correlations were revealed in ROC statistics. In survival analysis, DSS was lower for patients with high entropy (median survival 25.0 vs. 64.6 months, p = 0.003, see Table 3 and Figure 2). The other textural parameters as well as ‘classical’ PET parameters showed no significant difference in the log-rank test. Similarly, none of the investigated parameters was significantly correlated with OS. Univariate Cox regression (see Table 4) showed significant associations of DSS with entropy, yielding a HR of 5.92 (CI 1.28–27.39, p = .023) and MTV with a HR of 1.06 (CI 1.01–1.12, p = .031). Multivariate Cox regression was executed with a restriction to three independent variables due to low event numbers. Textural parameters were tested against MTV as the best ‘classical’ parameter, tumor size measured in CT and cumulative dose; similarly, MTV was tested against entropy and cumulative dose. Results showed only entropy as a significant, independent factor on DSS with a HR of 7.48 (CI 1.45–38.7, p = .016). Overall survival was again not associated with any PET parameter.

**Table 3:** **Kaplan-Meier analysis of overall and disease-specific survival**

**Parameter**	**Time to local recurrence (T2)**							**Overall survival**					**Disease-specific survival**					
	**Cutoff**	**Low risk**		**High risk**		**P**	**Cutoff**	**Low risk**		**High risk**		**P**	**Cutoff**	**Low risk**		**High risk**		**P**
		**n**	**m**	**n**	**m**			**n**	**m**	**n**	**m**			**n**	**m**	**n**	**m**	
SUV_max_	9.64	0	-	7	19.4	.052	11.2	12	36.0	16	25.2	.161	12.6	4	56.8	8	35.1	.080
SUV_mean_	4.52	2	52.9	5	48.7	.459	5.02	15	33.7	13	26.7	.283	5.39	6	53.7	6	36.3	.296
Entropy	**6.98**	**0**	**-**	**7**	**16.8**	**.001**	6.83	11	38.3	17	24.8	.111	**6.85**	**2**	**64.6**	**10**	**25.0**	**.003**
Correlation	**.453**	**3**	**61.0**	**4**	**10.6**	**.004**	.228	10	39.3	18	25.7	.241	.319	5	55.5	7	34.0	.134
Contrast	231	3	44.8	4	53.1	.980	246	15	28.8	13	31.9	.790	222	4	47.1	8	46.6	.472
Busyness	**.562**	**1**	**65.8**	**6**	**40.2**	**.042**	.516	6	38.7	22	28.0	.338	.579	6	52.7	6	25.7	.081
Coarseness	.037	2	61.2	5	43.0	.193	.048	9	37.5	19	26.8	.338	.032	6	52.7	6	25.7	.081
COV	.522	1	52.8	6	50.3	.579	.669	14	34.0	14	26.6	.411	.527	4	56.5	8	35.5	.101
MTV	44.5	2	61.9	5	19.4	.111	18.0	6	43.4	22	28.3	.436	23.1	4	53.6	8	42.7	.151
CT diameter	39.0	2	58.0	5	48.6	.575	32.5	10	34.6	18	28.0	.235	32.5	3	54.0	9	43.0	.104

n - number of events; m - mean survival.

Significant values are printed in bold.

**Table 4:** **Effects of variables on OS and DSS**

**Parameter**	**Overall survival**				**Disease-specific survival**			
	**Univariate**		**Multivariate**		**Univariate**		**Multivariate**	
	**HR**	**p**	**HR**	**p**	**HR**	**P**	**HR**	P
Age	1.07 (0.70–1.65)	.746			0.78 (0.42–1.44)	.424		
SUV_max_	0.97 (0.72–1.29)	.819			1.06 (0.74–1.51)	.761		
SUV_mean_	1.00 (0.31–3.25)	.994			1.71 (0.37–7.59)	.480		
High entropy	1.85 (0.85–4.00)	.120	1.79 (0.70–4.59)‡	.225	**5.92 (1.28**–**27.4)**	**.023**	**9.52 (1.39**–**65.1)**‡	**.022**
High correlation	1.58 (0.72–3.47)	.250			2.35 (0.74–7.44)	.146	1.61 (0.44–5.90)‡	.474
High contrast	0.90 (0.43–1.92)	.793			1.56 (0.46–5.30)	.476		
High busyness	1.55 (0.62–3.84)	.347			2.70 (0.65–8.56)	.093	1.29 (0.14–12.2)‡	.822
High coarseness	0.68 (0.30–1.52)	.346			0.37 ( 0.12–1.18)	.093	0.77 (0.08–7.28)‡	.822
High COV	1.52 (0.71–3.25)	.278			2.65 (0.79–8.91)	.114	1.98 (0.52–7.53)‡	.401
MTV*	1.02 (0.99–1.06)	.223	1.55 (0.27–8.95)†	.623	**1.06 (1.01**–**1.12)**	**.031**	2.11 (0.12–38.1)†	.613
CT diameter	1.01 (0.98–1.04)	.555			1.03 (0.98–1.07)	.245		

†tested against entropy, cumulative dose and CT diameter ‡tested against MTV, cumulative dose and CT diameter.

*analyzed on log scale.

Significant values are printed in bold.

### Stability and size-dependency

**Figure 3:**
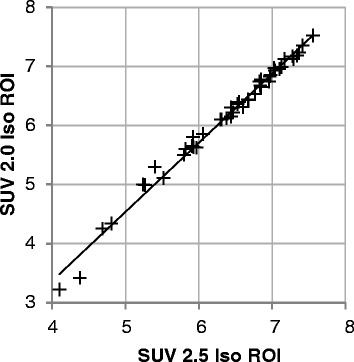
Influence of tumor segmentation. Correlation between entropy values calculated on SUV 2.0 and SUV 2.5 isocontour VOIs.

Calculations of the textural parameters described above were executed on VOIs based on SUV 2.0 isocontours. In order to estimate their stability with regard to VOI selection, they were also determined for a SUV 2.5 isocontour. Parameter ranges, correlation coefficients and levels of significance are shown in Table 5. The results demonstrate highly significant correlations between the two VOI definitions for all investigated parameters, with highest r values for SUV mean (r = .997), COV (r = .996) and entropy (.996; all p < 0.001; see Figure 3). Naturally, SUV max was independent of the isocontour used (r = 1).

**Table 5:** **Correlation of parameters calculated on SUV 2.0 and SUV 2.5 Iso ROIs**

	**SUV 2.0**		**SUV 2.5**		**r**	**P**
	**Range**	**Mean ± SD**	**Range**	**Mean ± SD**		
SUV_max_	3.85-43.1	13.2 ± 6.74	3.85-43.1	13.2 ± 6.74	1	.000
SUV_mean_	2.44-11.0	5.06 ± 1.66	3.00-12.4	5.71 ± 1.79	.997	<.001
MTV	5.77-204	42.1 ± 46.8	1.74-178	33.9 ± 39.5	.989	<.001
COV	.188-.933	.513 ± .144	.132-.839	.438 ± .138	.996	<.001
Entropy	4.58-7.52	6.53 ± .76	4.10-7.56	6.38 ± .84	.996	<.001
Correlation	.032-.555	.255 ± .159	-.126-.515	.189 ± .152	.963	<.001
Contrast	13.3-411	234 ± 110	12.2-395	226 ± 105	.990	<.001
Busyness	.432-.708	.557 ± .071	.370-.692	.544 ± .072	.933	<.001
Coarseness	.007-.104	.044 ± .025	.009-.158	.050 ± .029	.813	<.001

SD - standard deviation.

In order to quantify a possible dependency on PET volume, the examined parameters were correlated with MTV (see Table 6). Results showed significant correlations for all textural parameters except contrast and COV, with highest r values for coarseness and busyness (r = 0.747 and 0.860). SUV_max_ and SUV_mean_ showed as well significant correlations with MTV (r = 0.341 and 0.318).

**Table 6:** **Correlation of SUV and textural parameters with MTV**

	**R**	**P**
SUV_max_	0.341	.022
SUV_mean_	0.318	.033
Entropy	0.544	<.001
COV	.213	.161
Correlation	0.513	<.001
Contrast	−0.056	.714
Busyness	0.860	<.001
Coarseness	0.747	<.001

Respiratory movement as another potential biasing factor in textural analysis was assessed in 8 out of 45 patients who received 4D-CT, yielding a medial movement amplitude of 4.7 ± 2.9 mm (see Table 1).

## Discussion

The aim of the present study was to determine whether textural analysis can provide additional predictive information regarding recurrence and survival in NSCLC patients receiving primary SBRT, compared to classical PET parameters like SUV_max_ and MTV. As a result, our data support a possible association between tumor heterogeneity measured by textural analysis using entropy, correlation, busyness and coarseness on baseline FDG PET and local recurrence. SUV_max_ and SUV_mean_, as classical quantitative PET parameters, were not predictive for LR, which has already been shown by other authors [10-12], although one study reported a different result [22]. Another recent publication showed significance of SUV_max_ for prediction of LR only when treated as a continuous variable in regression analysis but not in the other statistical tests used [9].

DSS was significantly linked to PET texture described by entropy, which is in accordance to earlier studies showing the predictive value of PET texture for advanced and early stage NSCLC patients undergoing combined radio-chemotherapy [18,17]. Maximum or mean SUV values were not predictive for DSS, contradicting results by Horne et al. [9], although those were acquired in a larger cohort. The correlation between overall survival and the examined PET parameters was also not significant, most probably due to the large number of deaths caused by concomitant co-morbidity (16 patients vs. 12 patients who succumbed to the tumor, see Table 1). Finally, no parameter was predictive for the occurrence of mediastinal lymph node or distant metastases alone, although there have also been reports on an association with SUV [9,10].

While the homogeneity of the investigated patient cohort in terms of tumor stage and treatment modality minimizes bias originating from these factors, the influence of tumor size is more complex. Although the employed textural features are designed to be relatively robust against changes in image resolution, we could demonstrate a correlation of several textural parameters to MTV, a dependency which had also been reported by other authors [23,17]. It should be mentioned here that also SUV_max_ and SUV_mean_ were significantly correlated to MTV which may be caused to some degree by partial volume effects [24]. Orhac et al. therefore conclude that an investigation of the influence of tumor size on the dependent variables is mandatory [25]. Additionally, in our study, tumor size alone was predictive for local failure; LR occurred solely in tumors measuring more than 3 cm. This association has been reported in earlier studies as well [21,26] and should be kept in mind as possibly undermining the correct interpretation of data on local failure in SBRT, perhaps originating from insufficient and/or inhomogeneous dose delivery to larger tumors [27]. However, when only tumors larger than 3 cm were examined (i. e. T2 tumors), texture as measured by the features entropy and correlation, but not MTV or tumor size in CT, were significantly correlated with LR. Additionally, we performed multivariate regression analysis with MTV and CT diameter as covariates for survival, showing entropy to be a size-independent predictor of DSS.

In our opinion, these results suggest that the described association between FDG-PET texture, recurrence and survival is not merely a size effect, but also points to differences in tumor biology. Not much is currently known about the nature of these differences, however. Increased heterogeneity in CT tumor images has been associated with regional variations in hypoxia and angiogenesis [28], and similar histologic studies should be performed for FDG texture. A correlation with other functional imaging modalities, such as advanced MRI (e. g. diffusion-weighted and perfusion imaging) may provide additional clues to the biology underlying textural PET features.

Regarding the choice of heterogeneity parameters, though, there are still open questions to be answered. The above-mentioned study by Cook et al. favored coarseness and busyness as best predictors of overall survival in the setting of radio-chemotherapy [18]. In our cohort, which differed in tumor extent (early stage tumors) and therapy (stereotactic radiation), these parameters were still predictive for local recurrence, but did not perform better than MTV and, while significant in univariate survival analysis, were not identified as independent predictors in multivariate analysis. Instead, local entropy, which has not been investigated by Cook et al., was the most favorable parameter. Heterogeneity determined by entropy has also been shown to be predictive in low dose CT scans of lung cancer [29] and has been demonstrated as being relatively robust when examining inter-study variability [30] and different methods of tumor segmentation [25]. This robustness was also confirmed in our cohort, where entropy showed a high correlation between different VOI definitions (based on SUV 2.0 vs. SUV 2.5 thresholds), comparable to SUV_mean_ and MTV. The features correlation and contrast values also showed a good stability, while r values for busyness and coarseness were slightly lower, but still significant. In our view, these results suggest that the examined textural parameters are stable enough to be of utility for assessing heterogeneity of lung tumors.

Possible bias in this work also originates from the influence of respiratory movement on textural measurements which has not yet been thoroughly evaluated. A recent study showed moderate differences between gated and non-gated acquisitions [31], but entropy and correlation were not included in that investigation, and similar results have been reported before for standard SUV values [32]. 8 out of 45 of our patients received 4D-CT enabling assessment of the movement amplitude which was found to be in the expected range. Overall, blurring from respiratory movement is not likely to have a major effect on the textural parameters determined in our study; however, respiratory gated PET may be principally advantageous over ungated measurements in determining tumor heterogeneity and should be encouraged for comparative reasons in future studies on PET texture in lung tumors.

Our study is limited in being retrospective and by its relatively small sample size. Higher case numbers and prospective studies will certainly be needed before textural analysis on FDG-PET of lung tumors can be incorporated into routine clinical practice.

## Conclusion

Our study adds to the growing evidence that tumor heterogeneity as described by FDG-PET texture is associated with response to radiation therapy in NSCLC. In the future, individual therapy planning may benefit from these results, by e.g. dose escalation in tumors prone to local recurrence or by addition of chemotherapy. However, retrospective design is a major limitation of all studies published on this topic up to now. Reproducibility in prospective trials with higher case numbers will be a prerequisite for the inclusion of PET texture into routine clinical practice.

## Abbreviations

AUC: Area under the curve
CI: Confidence interval
COV: Coefficient of variation
CT: Computed tomography
DM: Distant metastases
DSS: Disease-specific survival
FDG: ^18^ F-fluorodeoxyglucose
GTV: Gross tumor volume
HR: Hazard ratio
LN: Lymph node metastases
LR: Local recurrence
MTV: Metabolic tumor volume
NSCLC: Non small cell lung cancer
OS: Overall survival
PET: Positron emission tomography
ROC: Receiver operating characteristic
SBRT: Stereotactic body radiation therapy
SUV: Standardized uptake value
SUV_max_: Maximum standardized uptake value
VOI: Volume of interest
